# Correction: Associations between age at natural menopause and risk of hypothyroidism among postmenopausal women from the Canadian Longitudinal Study on Aging (CLSA)

**DOI:** 10.1371/journal.pone.0338845

**Published:** 2025-12-15

**Authors:** The PLOS One Staff

## Notice of Republication

This article was republished on November 19, 2025, to correct errors in the Data Availability statement The correct statement is: Data are available from the Canadian Longitudinal Study on Aging (www.clsa-elcv.ca) for researchers who meet the criteria for access to de-identified CLSA data.

There are errors in the acknowledgement section. The acknowledgement section should read: This research was made possible using the data/biospecimens collected by the Canadian Longitudinal Study on Aging (CLSA). This research has been conducted using the CLSA Baseline Tracking Dataset Version 4.0, Baseline Comprehensive Dataset Version 7.0, Follow-up 1 Tracking Dataset Version 2.3, Follow-up 1 Comprehensive Dataset Version 3.2, Follow-up 2 Tracking Dataset Version 1.0, Follow-up 2 Comprehensive Dataset Version 1.0 and participant status version 3.0, under Application Number 2206014. The CLSA is led by Drs. Parminder Raina, Christina Wolfson and Susan Kirkland.

The opinions expressed in this manuscript are the author’s own and do not reflect the views of the Canadian Longitudinal Study on Aging.

## Supporting information

S1 File Originally published, uncorrected article. (PDF)

S2 File Republished, corrected article. (PDF)
