# Supplementary material for: Correction: Associations between age at natural menopause and risk of hypothyroidism among postmenopausal women from the Canadian Longitudinal Study on Aging (CLSA)
Source: PLoS One. 2025 Dec 15;20(12):e0338845. doi: 10.1371/journal.pone.0338845 (PMC12704873; doi:10.1371/journal.pone.0338845)
Supplement: S1 File — (PDF) [file pone.0338845.s001.pdf]

PLOS One

RESEARCH ARTICLE

Associations between age at natural menopause and risk of hypothyroidism among postmenopausal women from the Canadian Longitudinal Study on Aging (CLSA)

Durmalouk Kesibi*, **Michael Rotondi**, **Heather Edgell**, **Hala Tamim**

School of Kinesiology and Health Science, York University, Toronto, Ontario, Canada

* durra-kesibi@hotmail.com

Check for updates

OPEN ACCESS

Citation: Kesibi D, Rotondi M, Edgell H, Tamim H (2025) Associations between age at natural menopause and risk of hypothyroidism among postmenopausal women from the Canadian Longitudinal Study on Aging (CLSA). PLoS One 20(5): e0324635. https://doi.org/10.1371/journal.pone.0324635

Editor: Silvia Naitza, Consiglio Nazionale delle Ricerche, ITALY

Received: October 29, 2024

Accepted: April 28, 2025

Published: May 28, 2025

Copyright: © 2025 Kesibi et al. This is an open access article distributed under the terms of the Creative Commons Attribution License, which permits unrestricted use, distribution, and reproduction in any medium, provided the original author and source are credited.

Data availability statement: Dr. Michael Rotondi (MR) is partially supported by the Canadian Institutes of Health Research (https://cihr-irsc.gc.ca/e/193.html) and the Public Health Agency of Canada (https://www.canada.ca/en/public-health.html) through the Applied

Abstract

Menopause is a key period in women's lives associated with major physiological changes. Early menopausal age has been linked to a range of adverse outcomes. Estrogen has been found to increase levels of thyroid binding proteins in the blood; however, its effect on hypothyroidism is not well investigated. To date limited studies were conducted to investigate the association between age at natural menopause and incidence of hypothyroidism, thus the objective of this study is to investigate the association between age at natural menopause and incidence of hypothyroidism among postmenopausal Canadian women. The study included women from the Canadian longitudinal study on aging that were followed for a 10-year period. Analysis was restricted to naturally postmenopausal women without hypothyroidism prior to menopause. Age at natural menopause was examined using the following categories 40–44, 45–49,50–54 (reference), and ≥55. Survival analysis was utilized to determine time to onset of hypothyroidism. Unadjusted and adjusted multivariable Cox regression models were used to assess the relationship between age at natural menopause and incidence of hypothyroidism. The multivariable Cox regression analysis showed no significant association between age at natural menopause and risk of hypothyroidism.

Introduction

Hypothyroidism is a condition where the thyroid gland does not make adequate amounts of thyroid hormones: Thyroxine (T4) and triiodothyronine (T3) [1]. Thyroid hormones are carried to all tissues in the body where they help metabolism, maintain thermoregulation, and sustain the function of the brain, heart, muscles, and other organs [2]. Some signs and symptoms of hypothyroidism include fatigue, weight gain, constipation, cold intolerance, depression, hair loss, bradycardia, and goiter [1]. The main cause of hypothyroidism in the developed world is autoimmune dysregulation

Public Health Chairs program (Award #PP8-192587). Michael Rotondi was involved in the supervision, formal analysis, writing -revision & editing of the manuscript. The Canadian Institutes of Health Research and the Public Health Agency of Canada had no role in study design, data collection and analysis, decision to publish, or preparation of the manuscript.

Funding: Dr. Michael Rotondi (MR) is partially supported by the Canadian Institutes of Health Research (https://cihr-irsc.gc.ca/e/193.html) and the Public Health Agency of Canada (https://www.canada.ca/en/public-health.html) through the Applied Public Health Chairs program (Award #PP8-192587). Funding for the Canadian Longitudinal Study on Aging (CLSA) (www.clsa-elcv.ca) is provided by the Government of Canada through the Canadian Institutes of Health Research (CIHR) under grant reference: LSA 94473 and the Canada Foundation for Innovation, as well as the following provinces, Newfoundland, Nova Scotia, Quebec, Ontario, Manitoba, Alberta, and British Columbia.

Competing interests: The authors have declared that no competing interests exist.

[1]. Hypothyroidism has been found to affect 4.6% of the US population [3]. The prevalence of hypothyroidism is three to seven times higher in women than men and the incidence increases with age [4]. A large study in the UK with twenty years of follow-up found an incidence of hypothyroidism of 0.6 per 1,000 person-years in males and 3.5 per 1,000 in females person-years [5].

Thyroid function is controlled by the hypothalamic-pituitary-thyroid-axis [6]. The anterior pituitary gland releases thyroid stimulating hormone (TSH), which stimulates the thyroid gland to secret thyroid hormones (T3 &T4) into the blood where they have a negative feedback effect on TSH [6]. Thyroid hormones can exist in a free or bound form. Only the free form can enter target tissues, while the other form is bound to thyroid binding globulin (TBG) - a protein which carries thyroid hormones in the blood [7]. Hypothyroidism is usually indicated by high TSH and low free T4 levels [8]. While subclinical hypothyroidism (a milder form of the disease) is indicated by an elevated TSH and normal free T4 [9].

Menopause is an integral period in women's lives marked by major physiological changes, including a significant drop of estrogen [10]. The average age at menopause is 51 years [10], and both menopause and hypothyroidism affect reproductive hormones [11]. It has been found that 70% of hypothyroidism cases are in patients over the age of 50 years at the time of diagnosis [12], with an increase of incidence in the postmenopausal period [13]. Further, subclinical hypothyroidism frequently exists or develops during menopausal transition and is mainly due to autoimmune dysfunction [6]. A recent study has found that 20–35% of women with premature ovarian failure (POF) have thyroid autoimmune diseases [14], and guidelines suggest that women with POF must be measured for thyroid antibodies [15]. This could suggest a role of early menopause in thyroid disease.

Few studies have investigated the incidence of hypothyroidism due to changes in reproductive factors. A study found that the incidence of hypothyroidism decreased during pregnancy but increased sharply in the postpartum period [16]. One study looked at age at menarche and found that early menarche was associated with an increased risk of subclinical hypothyroidism; however, it was a cross-sectional study [17]. There is contradicting evidence about the effect of oral contraceptives use and risk of hypothyroidism. A study evaluating the long-term use of oral contraceptives found increased risk of hypothyroidism (OR 4.71; 95% CI 1.7–12.9) after adjusting for sociodemographic, health-related and reproductive factors [4] and additional studies have found that current use is associated with an increased incidence [18]. However, other studies have found no significant association between ever and current use of oral contraceptives [19,20], yet these studies do not specify the type of oral contraceptives used.

Earlier age at menopause has been found to increase risk of several autoimmune and endocrinological diseases [21–23]. Only two studies have examined the association of age at menopause and the subclinical form of hypothyroidism, and they have shown contradicting results [6,17]. Overall, no study has examined the effect of age at natural menopause (ANM) on risk of hypothyroidism in a longitudinal design. Some studies have shown that the presence of estrogen could increase thyroid disorders

by increasing thyroid cell proliferation and leading to conditions like goiter [24]. This study will examine the association between ANM and incidence of hypothyroidism among postmenopausal women from the Canadian Longitudinal Study on Aging (CLSA).

Methods

Study design and sample

This study involved a secondary data analysis from CLSA. CLSA is a Canada wide study of 51,338 males and females between the ages of 45–85 years at recruitment over a 20-year period with the aim of understanding factors associated with the well-being of the aging population. CLSA consists of two cohorts: Tracking and Comprehensive. At baseline, the Tracking cohort included 21,241 participants (male: 10,406; female: 10,835), selected randomly from across the 10 Canadian provinces and interviewed by telephone. The Comprehensive cohort included 30,097 participants (male:14,777 & female: 15,320) who were randomly selected from 7 of the 10 Canadian provinces and had to be within 25–50 km from one of the 11 data collection sites. Data collection for this cohort involved an in-person home interview and more in-depth data collection at one of the data collection sites. CLSA data collection occurs every three years, with three cycles of data currently available (baseline, follow-up 1, and follow-up 2). Approximately 21.42% of the sample was lost to follow-up or had died by follow-up 2. More details on CLSA method can be found elsewhere [25,26].

This study was a retrospective analysis of de-identified data accessed from CLSA on November 10, 2022. It included combined Tracking and Comprehensive cohorts, including three cycles of data collection (baseline, follow-up 1, and follow-up 2). The CLSA study design excluded residents of the Canadian territories, remote regions, Federal First Nations reserves, and other provincial First Nations settlements. It also excluded full-time members of the Canadian Armed Forces, individuals living in institutions, those unable to respond in English or French, and those who are cognitively impaired at recruitment. The CLSA study has been approved by McMaster University Health Integrated Research Ethics Board and by research ethics boards at all collaborating Canadian institutions. This study is a secondary data analysis of fully de-identified CLSA data approved by the York University, Office of Research Ethics (ORE) [STU 2022-114]. Further consent from participants was not required as all CLSA participants provided informed consent during primary data collection to have their de-identified data used in future research.

Study participants

All males were excluded, leaving 26,155 females in the study. Other exclusions included: women with missing information on menopause, women that did not reach menopause, women with surgical or medically induced menopause, women with missing age at natural menopause, women with age at menopause under 40 years or over 67 years, similar to cut-offs proposed by Verschoor and Tamim [27]. The study also excluded women with missing information on hypothyroidism incidence, and women with hypothyroidism prior to age at natural menopause. A participant flow diagram and common exclusions are shown in Fig 1.

Exposure assessment (age at natural menopause (ANM))

Self reported age at natural menopause was ascertained from all three cycles (baseline, follow-up1 & follow-up2) with the question: "*Have you gone through menopause, meaning that your menstrual periods stopped for at least one year and did not restart?*" answers were recorded as, "Yes" or "No". Women who answered "Yes" were then asked about their age at menopause: "*How old were you when your menstrual periods stopped for at least one year and did not re-start?*" answers were reported in years of age. ANM was categorized into the following groups: 4044, 45–49,50–54 (reference), and ≥55, similar to categories presented in Mondul et al. [28] and Brand et al. [21]. Having ANM as categorical variable allows for better detection of non-linear relationships.

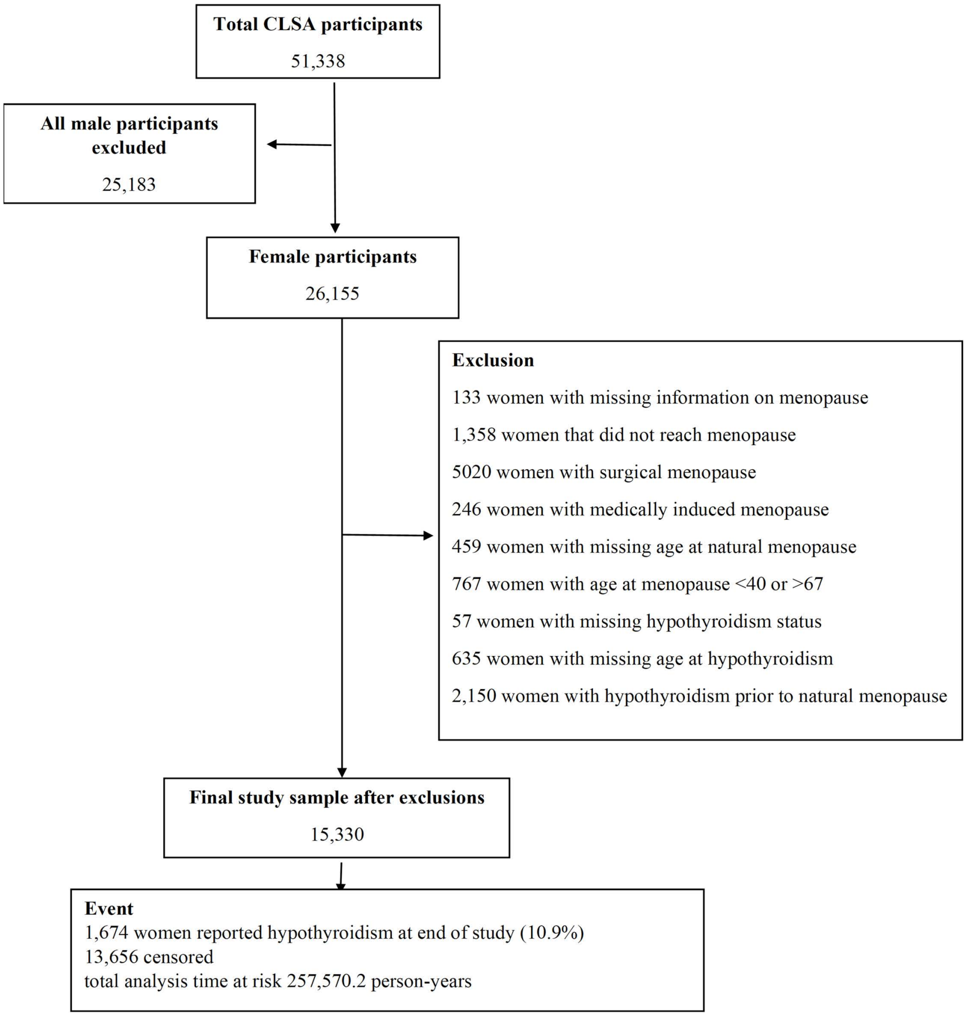

Fig 1. Canadian Longitudinal Study on Aging (CLSA) participant chart.

https://doi.org/10.1371/journal.pone.0324635.g001

Outcome assessment (incidence of hypothyroidism)

Information on incidence of hypothyroidism following menopause was collected using the following questions. First, women were asked, "*Has a doctor ever told you that you have an UNDER-active thyroid gland (sometimes called hypothyroidism or myxedema)?answered by "*Yes" or "No". Women who answered by "Yes" were asked about their age of diagnosis: "*At what age, or in what year, were you first told you had hypothyroidism?*" answers were reported in years of age. Women who answered by "No" were asked in the next cycle. The Incidence of hypothyroidism was collected from all three cycles in the Comprehensive cohort and from follow-ups 1 & 2 cycles only of the Tracking cohort because information on age at hypothyroidism is not available from baseline Tracking.

Covariates

Sociodemographic factors included: Ethnicity (White, other), where "other" included, South Asian, Chinese, Filipino, Latin American, Japanese, Southeast Asian, Korean, Arab, West Asian, Black, and other North American origins, and education level (less than high school, high school to some college, bachelor's degree or higher). Health-related factors included:

Smoking status (never, former, current), alcohol consumption (never, less than once weekly, at least once weekly), frequency of leisure time physical activity in the past year (nonregular, regular), with regular defined as participation in physical activity at least once a week. Height and weight were used to calculate body mass index (BMI) in kg/m^2. In the Tracking cohort information to determine BMI was self-reported while in the Comprehensive cohort it was measured. The BMI cut-off for under weight was increased from the standard 18.5 kg/m^2 because very few participants had that low of a BMI [29]. BMI level was categorized as, underweight < 20.0 kg/m^2, normal weight = 20.0–24.99 kg/m^2, overweight = 25.0–29.99 kg/m^2, and obese >30 kg/m^2. Reproductive factors were also adjusted for and included age at menarche in years (≤11, 12–14, ≥ 15), duration of oral contraceptives in years (0–3,4–7,8–11, ≥ 12), number of births (0,1,2,3, ≥ 4), duration of hormone replacement therapy (HRT) in years (never, < 1,1–2,3–4, ≥ 5), and HRT type (none, combined estrogen and progesterone, estrogen, and progestrone). All covariates were measured at baseline, except for number of births which was obtained from follow-up 1, age at menarche which was obtained from follow-up 2, and duration of oral contraceptives which was obtained from follow-ups 1 & 2.

Endpoints

Women were followed for 10 years and the incidence of hypothyroidism after menopause was the primary outcome measure. Women who did not develop hypothyroidism during the study period were considered censored observations but contributed person-years. The end of follow-up was defined at the earliest occurrence of one of the following events: a) incidence of hypothyroidism b) loss to follow-up or death or c) end of the study period.

Statistical analysis

Survival analysis was utilized to determine time to onset of hypothyroidism. Descriptive statistics by ANM categories were used to describe the sample. The data are presented in frequencies and percentages and compared using a chi-square test. Missing data was addressed using multiple imputation by chained equations (MICE). Three variables, which were not collected at baseline but at later cycles (Oral contraceptive duration, number of births, and age at menarches), were found to have a moderate amount of missingness (17.6%, 13.5%, 22.8%, respectively), leading us to adopt a missing-not-at-random and multiple imputation strategy. We applied the MICE procedure using the 'mi impute chained' command in Stata version 18 to all variables [31]. Specifically, we generated 20 imputed data sets, using logistic regression for binary variable and ordered logistic regression for categorical variable. Regression coefficients were combined using Rubin's rule [30]. Kaplan-Meier survival curves were used for the bivariate analysis to determine time-onset of hypothyroidism, and a log-rank test was used to compare the survival curves. Unadjusted and adjusted cox proportional hazard regression models were used to estimate the hazard ratios (HR) and their 95% confidence intervals (CIs) for the association between ANM (in years: 40–44, 45–49, 50–54 (reference), or ≥55) and incidence of hypothyroidism. BMI and HRT were considered in the model as interaction terms with ANM and no significance was found. The Cox proportional hazard assumption was evaluated using log-log plots. Inverse probability weights provided by the CLSA were used to make results generalizable to the Canadian population [31]. Inverse probability weights provided by the CLSA were used to make results generalizable to the Canadian population. Inflation weights were used for descriptive statistics and analytics weights were used for regression analysis. A p-value of 0.05 was considered statistically significant. All statistics were calculated using Stata statistical software (version 18, StataCorp LLC, College Station, TX, USA).

Results

This study included 15,330 postmenopausal women (weighted N = 4,009,706). Exclusions are presented in Fig 1. During the study's follow-up period 1,674 (10.9%) reported hypothyroidism incidence with a follow-up time of 257,589.7 person-years, resulting in a hypothyroidism incidence of 6.5 per 1000 person-years. The mean age at baseline was 60.6 years. The mean and median ANM were 50.3(SD: 4.3) and 50 (IQR: 48–53)years, respectively, while the median

age at hypothyroidism diagnosis was 60 years. Over half of the sample was between 45–64 years (68.5%) at baseline. Most of the sample was of White origin (94.4%). Over half of the sample had high school to some college education (62.5%). Only 12.6% of the sample were current smokers, and 12.5% reported ever having cancer. Around half the sample consumed alcohol more than once weekly (48.1%) and engaged in regular physical activity (52.8%). Normal BMI range was found in 36.8% of the sample. Oral contraceptive use of 12 years or more was found in 22.7% of the sample. the most common number of births was two children per family (34.4%). A large portion of the sample never used HRT (71.1%), and half of the sample had average age at menarche of 13 years (52.9%). Table 1 shows characteristics of the study population categorized by ANM. ANM differed on several sociodemographic, health-related, and reproductive factors.

Fig 2 shows the bivariate analysis using Kaplan-Meier survival curves depicting time to onset of hypothyroidism for each ANM category. The estimated p-value was 0.7, suggesting no evidence of a significant difference between ANM categories and incidence of hypothyroidism. Table 2 shows the unadjusted and adjusted multivariable cox regression model with HRs and their 95% CIs. No evidence of any significant associations between ANM and risk of hypothyroidism was found. Women with BMI levels between 25–29 and equal to or more than 30 showed an increased risk of hypothyroidism with HR of 1.2 (95% CI: 1.1–1.4) and HR of 1.4 (95% CI: 1.2–1.6), respectively. Fig 3 shows the Cox proportional hazard assumption using log-log plots. Lines on the plot are overlapping indicating a violation of the proportional hazard assumption. Robust standard errors were used to ensure that the model remained robust against potential violations of the proportional hazard's assumption.

Discussion

This study examined the association between ANM and risk of hypothyroidism over a 10-year period among postmenopausal women. The multivariable Cox regression model found no evidence of a statistically significant association between ANM and incidence of hypothyroidism. Although no previous study has examined the association between ANM and hypothyroidism, we can compare our results to studies that looked at subclinical hypothyroidism, a milder form of hypothyroidism. Our results align with findings of a cross-sectional study by Kotopouli et al., (2019), which reported no significant difference in age at menopause in women with or without subclinical hypothyroidism [17]. In contrast, a cross-sectional study by Monterrosa-Castro et al. [14] on 643 Colombian women found increased likelihood of subclinical hypothyroidism in women with age at menopause between 40–44 with an odds ratio (OR) of 3.37 (95% CI 1.40–8.10), and in women with age at menopause before 40 years with an OR of 4.31 (95% CI 1.24–14.97). The study also reported an increased risk in the combined group of age at menopause of less than 45 years with an OR of 3.57 (95% CI 1.57–8.10), compared to women with age at menopause of 45 years or older [14]. However, the study was focused on Colombian women and did not account for the average age at menopause which is typically between 50–54 years. The study also included older age at menopause of up to 69 years in the reference and included women with surgical menopause, which has been found to increase risk of thyroid cancer [6]. These differences in the reference group, inclusion criteria, and the authors' use of a cross-sectional study design, could explain the contrasting findings.

Estrogen has been found to negatively affect women with existing hypothyroidism by significantly increasing hypothyroidism markers [32,33]. Studies of estrogen administration in women without existing hypothyroidism found no significant changes in hypothyroidism markers [7,34] or found a significant increase in hypothyroidism markers that remained within normal ranges [35,36]. Estrogen's effect on hypothyroidism is mainly due to its ability to increase TBG levels. Studies on HRT and hypothyroidism markers showed a significant increase in TBG levels after estrogen administration, this was not necessarily observed for combined therapy [7,32,33,35,36]. Estrogen increases TBG levels through reducing its clearance and enhancing its biosynthesis [32]. Higher TBG levels in the blood lead to an increase of its binding with free T4 leading to slower entry of T4 into cells. This signals for more production of TSH which

Table 1. Characteristics of study population according to age at natural menopause.

	Age at Natural Menopause								
	40-44		45-49		50-54		≥ 55		P-value [a]
**Variables**	**Unweighted**	**Weighted[a]**	**Unweighted**	**Weighted[a]**	**Unweighted**	**Weighted[a]**	**Unweighted**	**Weighted[a]**	
N (%)	1,263 (8.2)	372021.3 (9.3%)	3,568 (23.3)	971274.8 (24.2%)	7,722 (50.4)	1980849.4 (49.4%)	2,777 (18.1)	685560.6 (17.1%)	
**Ethnicity**									
White	1,199 (94.9%)	358973.0 (96.5%)	3,344 (83.7%)	914957.8 (94.2%)	7,303 (94.6%)	1853987.9 (93.6%)	2,653 (95.5%)	656548.6 (95.8%)	0.0585
Other[a]	52 (4.1%)	10665.7 (2.9%)	168 (4.7%)	37955.6 (3.9%)	331 (4.3%)	98935.9 (5.0%)	98 (3.5%)	18347.2 (2.7%)	
**Education Level**									
Less than high school	120 (9.5%)	91559.1 (24.6%)	231 (6.5%)	198500.1 (20.4%)	428 (5.5%)	325897.5 (16.5%)	160 (5.8%)	131102.6 (19.1%)	<0.001
High school – some college	780 (61.8%)	240379.6 (64.6%)	2,0340 (57.0%)	598812.94 (61.7%)	4,101 (53.1%)	1247791.2 (63.0%)	1,499 (54.0%)	420812.7 (61.4%)	
Bachelor's or higher	355 (28.1%)	37237.1 (10.0%)	1,294 (36.3%)	169293.6 (17.4%)	3,180 (41.2%)	404729.1 (20.4%)	1,113 (40.1%)	131595.8 (19.2%)	
Health-related Factors									
**Smoking**									
Never	394 (31.2%)	98894.8 (26.6%)	1,104 (30.9%)	251546.3 (25.9%)	2,804 (36.3%)	665611.5 (33.6%)	1,019 (36.7%)	253527.0 (37.0%)	<0.001
Current	198 (15.7%)	75910.8 (20.4%)	461 (12.9%)	184735.3 (19.0%)	614 (8.0%)	201397.8 (10.2%)	614 (8.0%)	43236.3 (6.3%)	
Former	666 (52.7%)	194252.1 (52.2%)	1,982 (55.6%)	528763.9 (54.4%)	4,265 (55.2%)	1105441.9 (55.8%)	4,265 (55.2%)	384050.7 (56.0%)	
**Alcohol consumption**									
Never	177 (14.0%)	46184.4 (12.4%)	435 (12.2%)	122198 (12.6%)	791 (10.2%)	234717.9 (11.4%)	307 (11.1%)	84836.6 (12.4%)	0.5064
Less than once weekly	476 (37.7%)	148328.0 (39.9%)	1,309 (36.7%)	370393.0 (38.1%)	2,619 (33.9%)	685143.4 (34.6%)	978 (35.2%)	236342.1 (34.5%)	
More than once weekly	566 (44.8%)	163299.8 (43.9%)	1,680 (47.1%)	442139.8 (45.5%)	4,053 (52.5%)	983352.5 (49.6%)	1,399 (50.4%)	340672.2 (49.7%)	
**Leisure time physical activity**									
Non-regular	620 (49.1%)	184645.0 (49.6%)	1,1612 (45.2%)	485788.5 (50.1%)	3,275 (42.2%)	909015.0 (45.9%)	1,131 (40.7%)	306902.3 (44.8%)	0.0027
Regular	638 (50.5%)	184209.4 (49.5%)	1,946 (54.5%)	483231.0 (49.7%)	4,437 (57.5%)	1070223.3 (54.0%)	1,641 (59.1%)	3780.28.3 (55.1%)	
**BMI (Kg/m^2)**									
<20.00 (underweight)	60 (4.8%)	8147.3 (4.9%)	185 (5.2%)	64558.1 (6.6%)	350 (4.5%)	91122.5 (4.6%)	115 (4.1%)	23985.8 (0.6%)	0.0298
20.0-24.99 (normal weight)	418 (33.1%)	127351.8 (34.2%)	1,274 (35.7%)	302902.2 (31.2%)	2,882 (37.3%)	763054.0 (38.5%)	907 (32.7%)	228406.0 (33.3%)	
25.0-29.99 (overweight)	429 (34.0%)	121776.7 (32.7%)	1,221 (34.2%)	302902.2 (31.2%)	2,589 (33.5%)	635393.1 (32.1%)	1,014 (36.5%)	255393.6 (37.2%)	
> 30.0 (obese)	340 (26.9%)	97155.8 (26.1%)	873 (24.5%)	240370.9 (24.7%)	1,860 (24.1%)	473272.3 (23.9%)	731 (26.3%)	175894.5 (25.7%)	
**Cancer**									
Yes	235 (18.6%)	63005.2 (16.9%)	507 (14.2%)	126764.3 (13.15)	1,018 (13.2%)	224387.7 (11.3%)	395 (14.2%)	88408.9 (12.9%)	0.0133
No	1,026 (81.2%)	308152.5 (82.8%)	3,054 (85.6%)	843804.7 (86.9%)	6,693 (86.7%)	1755792.3 (88.6%)	2,377 (85.6%)	596854.6 (87.15%)	

(Continued)

Table 1. (Continued)

	Age at Natural Menopause								
	40-44		45-49		50-54		≥ 55		P-value [a]
Variables	Unweighted	Weighted[a]	Unweighted	Weighted[a]	Unweighted	Weighted[a]	Unweighted	Weighted[a]	
Reproductive Factors									
**Duration of Oral Contraceptive (years)**									
0-3	423 (33.5%)	106675.2 (28.7%)	1,148 (32.2%)	287192.2 (29.6%)	2,591 (33.6%)	645990.8 (32.6%)	990 (35.7%)	244338.6 (35.6%)	<0.001
4-7	174 (13.8%)	41944.4 (14.0%)	466 (13.1%)	119490.9 (12.3%)	1,226 (15.9%)	312997.1 (15.8%)	404 (14.6%)	97067.0 (14.2%)	
8-11	145 (11.5%)	42448.3 (11.4%)	453 (12.7%)	136796.3 (14.1%)	995 (12.9%)	275277.0 (13.9%)	360 (13.0%)	71849.3 (10.5%)	
≥12	229 (18.1%)	72698.1 (19.5%)	768 (21.5%)	316667.4 (22.3%)	1,642 (21.3%)	460801.5 (23.3%)	553 (19.9%)	160751.2 (23.45%)	
**Number of births**									
0	210 (16.6%)	54105.6 (14.5%)	640 (17.9%)	137635.5 (14.2%)	1,287 (16.7%)	267574.3 (13.5%)	343 (12.4%)	64172.0 (93.6%)	<0.001
1	165 (13.1%)	37178.4 (10.%)	402 (11.3%)	90622.8 (9.3%)	945 (12.2%)	221588.3 (11.25%)	324 (11.7%)	69835.8 (10.2%)	
2	375 (29.7%)	115788.6 (31.1%)	1,097 (30.8%)	308150.5 (31.7%)	2,630 (34.1%)	707586.7 (35.75%)	989 (35.6%)	246396.4 (35.9%)	
3	194 (15.4%)	56711.5 (15.2%)	634 (17.8%)	167612.8 (17.3%)	1,388 (18.0%)	379652.1 (19.2%)	548 (19.7%)	126245.8 (18.4%)	
≥4	124 (9.8%)	28298.9 (7.6%)	336 (9.4%)	104249.7 (10.7%)	766 (9.9%)	184576.6 (9.3%)	338 (12.2%)	101145.2 (14.8%)	
**Age at Menarche**									
≤11	192 (15.2%)	56931.7 (15.3%)	476 (13.3%)	129317.0 (13.3%)	1,070 (13.9%)	264808.7 (13.4%)	350 (12.6%)	87658.2 (12.8%)	<0.001
12-14	612 (48.5%)	158552.4 (42.6%)	1,951 (54.7%)	486512.88 (50.1%)	4,580 (59.3%)	1122828.5 (56.7%)	1,624 (58.5%)	351505.8 (51.3%)	
≥15	127 (10.1%)	36778.9 (9.9%)	326 (9.1%)	95682.9 (9.8%)	720 (9.3%)	212485.0 (10.7%)	315 (11.3%)	93660.3 (13.7%)	
**Duration of use of any HRT (years)**									
Never	659 (52.2%)	223925.7 (60.2%)	2,274 (63.7%)	661035.6 (68.1%)	5,458 (70.7%)	1482563.5 (74.8%)	1,899 (68.4%)	483370.9 (70.5%)	<0.001
<1	71 (5.6%)	17781.2 (4.8%)	206 (5.8%)	53912.6 (5.6%)	412 (5.3%)	90719.2 (4.6%)	145 (5.2%)	40234.8 (5.9%)	
1-2	102 (8.1%)	23889.7 (6.4%)	246 (6.9%)	53049.0 (5.5%)	460 (5.9%)	120403.2 (60.8%)	199 (7.2%)	40523.2 (5.9%)	
3-4	49 (3.9%)	14584.1 (3.9%)	143 (4.0%)	43127.3 (4.4%)	305 (4.0%)	65117.2 (3.3%)	113 (4.1%)	23619.3 (3.4%)	
≥5	370 (29.3%)	88301.0 (23.7%)	667 (18.7%)	153043.8 (15.8%)	1,037(13.4%)	211434.7 (10.7%)	393 9(14.2%)	92425.7 (13.5%)	
**Type of HRT**									
None	659 (52.2%)	223925.7 (60.2%)	2,274 (63.7%)	661035.6 (68.1%)	5,458 (70.7%)	1482563.5 (74.8%)	1,899 (68.4%)	483370.9 (70.5%)	<0.001
estrogen & progesterone	228 (18.1%)	50362.9 (13.5%)	538 (15.1%)	119874.8 (12.3%)	1,034 (13.4%)	204445.7 (10.3%)	368 (13.3%)	76864.1 (11.2%)	
Estrogen	241 (19.1%)	67869.6 (18.2%)	489 (13.7%)	119474.2 (12.3%)	727 (9.4%)	169287.7 (8.5%)	279 (10.1%)	69314.573 (10.1%)	

(Continued)

Table 1. (Continued)

	Age at Natural Menopause								
	40-44		45-49		50-54		≥ 55		P-value [a]
Variables	Unweighted	Weighted[a]	Unweighted	Weighted[a]	Unweighted	Weighted[a]	Unweighted	Weighted[a]	
Progestrone	35 (2.8%)	9322.5 (2.5%)	76 (2.1%)	25648.4 (2.6%)	171 (2.2%)	41700.6 (2.1%)	58 (2.1%)	10842.2 (2.2%)	

BMI, body mass index; CAD, Canadian dollars; 95% CI, 95% confidence intervals; HRT, hormone replacement therapy

All variables were obtained from baseline expect for number of births which was obtained from follow-up 1, age at menarche which was obtained from follow-up 2, and duration of oral contraceptives which was obtained from follow-ups 1 & 2.

[a]Estimated using inflation weights.

[b]Other included South Asian, Chinese, Filipino, Latin American, Japanese, Southeast Asian, Korean, Arab, West Asian, Black, and Other North American Origins.

Table is showing column percentages. Totals may not sum to 100% due to missing data.

https://doi.org/10.1371/journal.pone.0324635.t001

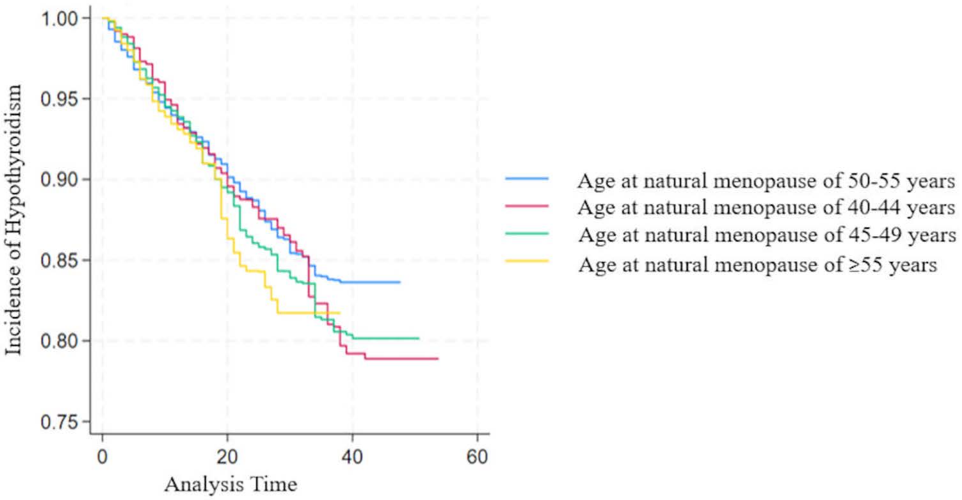

Fig 2. Kaplan Miere survival curves by ANM groups.

https://doi.org/10.1371/journal.pone.0324635.g002

consequently leads to an increase of the production of thyroid hormones [37]. It seems that this cycle increases levels of bound T4 but not free T4 thus not affecting hypothyroidism because healthy women are able to produce free T4 to compensate. However, women with existing hypothyroidism are not able to compensate for this effect and thus are at an increased risk of increased disease severity [32].

Hypothyroidism is correlated with higher BMI and obesity in our study and in others [38,39]. Two studies have shown a significant positive association between higher BMI levels and higher TSH levels [40,41]. Thyroid hormones play an important role in regulating thermogenesis, energy consumption, enzymes involved in lipid metabolism, and salt and water retention [42]. Moreover, a small increase in TSH has been found to be associated with deficiency in resting energy consumption [42]. Furthermore, low levels of thyroid hormones may lead to fat accumulation and reduced lipolysis [43]. This suggests that obesity in hypothyroid women may be a consequence of the condition rather than a cause.

Table 2. Unadjusted and adjusted hazard ratios (HRs) and 95% confidence intervals (CI) between age at natural menopause and risk of hypothyroidism.

Variables	Unadjusted HR (95% CI)[a]	Adjusted HR (95% CI)[ab]
**Age at Natural menopause (years)**		
40-44	0.9 (0.8-1.1)	0.9 (0.8-1.1)
45-49	1.0 (0.9-1.2)	1.0 (0.9-1.1)
50-54 (ref)	1	1
≥55	1.1 (0.9-1.3)	1.1 (0.9-1.2)
Sociodemographic		
**Ethnicity**		
White	1	1
Other[c]	1.1 (0.8-1.4)	1.1 (0.9-1.4)
**Education Level**		
Less than high school	0.8 (0.7-1.0)	0.8 (0.7-1.0)
High school – some college	0.9 (0.9-1.0)	0.9 (0.8-1.0)
Bachelors or higher	1	1
Health-related Factors		
**Smoking**		
Never	1	1
Current	0.8 (0.6-1.0)	0.8 (0.7-1.0)
Former	1.1 (0.9-1.2)	1.1 (1.0-1.2)
**Alcohol consumption**		
Never	1	1
Less than once weekly	1.2 (0.9-1.3)	0.8 (0.9-1.3)
More than once weekly	1.0 (0.9-1.2)	1.0 (0.8-1.2)
**Physical activity**		
Non regular	1	1
regular	1.0 (0.9-1.1)	1.00 (0.9-1.1)
**BMI (Kg/m^2)**		
<20.00 (underweight)	1.0 (0.8-1.3)	1.0 (0.8-1.3)
20.0-24.99 (normal weight)	1	1
25.0-29.99 (overweight)	**1.2 (1.1-1.4)**	**1.2 (1.1-1.4)**
> 30.0 (obese)	**1.4 (1.2-1.5)**	**1.4 (1.2-1.6)**
**Cancer**		
No	1	1
yes	1.1 (0.9-1.2)	1.0 (0.9-1.2)
Reproductive Factors		
**Duration of Oral Contraceptive (years)**		
0-3	1	1
4-7	1.0 (0.9-1.2)	1.0 (0.9-1.2)
8-11	1.0 (0.9-1.2)	1.0 (0.9-1.2)
≥12	1.0 (0.8-1.1)	1.0 (0.8-1.1)
**Number of births**		
0	1	1
1	1.0 (0.8-1.2)	1.0 (0.8-1.2)
2	1.0 (0.8-1.1)	1.0 (0.8-1.1)
3	0.9 (0.8-1.1)	1.0 (0.8-1.1)
≥4	1.0 (0.8-1.2)	1.0 (0.9-1.2)

(Continued)

Table 2. (Continued)

Variables	Unadjusted HR (95% CI)[a]	Adjusted HR (95% CI)[ab]
**Age at Menarche**		
≤11	1.0 (0.9-1.2)	1.0 (0.9-1.2)
12-14	1	1
≥15	1.0 (0.8-1.2)	1.0 (0.9-1.2)
**Duration of use of any HRT (years)**		
Never	1	1
<1	1.0 (0.8-1.3)	1.0 (0.7-1.4)
1-2	1.1 (0.9-1.3)	1.1 (0.8-1.5)
3-4	1.2 (0.9-1.5)	1.1 (0.8-1.6)
≥ 5	**1.2 (1.1-1.4)**	1.2 (0.9-1.6)
**Type of HRT**		
None	1	1
Combined estrogen and progesterone	1.2 (1.0-1.3)	1.1 (0.8-1.5)
Estrogen	1.2(1.0-1.3)	1.1 (0.8-1.5)
Progestrone	1.1 (0.8-1.5)	1.0 (0.6-1.5)

BMI, body mass index; CAD, Canadian dollars; CES-D, the Center for Epidemiological Studies Depression Scale; 95% CI, 95% confidence intervals; HR, hazard ratio; HRT, hormone replacement therapy.

[a]Calculated HR and 95% CI using survey analytical weights and robust standard errors

[b]Other included South Asian, Chinese, Filipino, Latin American, Japanese, Southeast Asian, Korean, Arab, West Asian, Black & other North American Origins.

Regression analysis was performed after implementing multiple imputation chained equation

Bold numbers indicate the significant results with p-value <0.05

https://doi.org/10.1371/journal.pone.0324635.t002

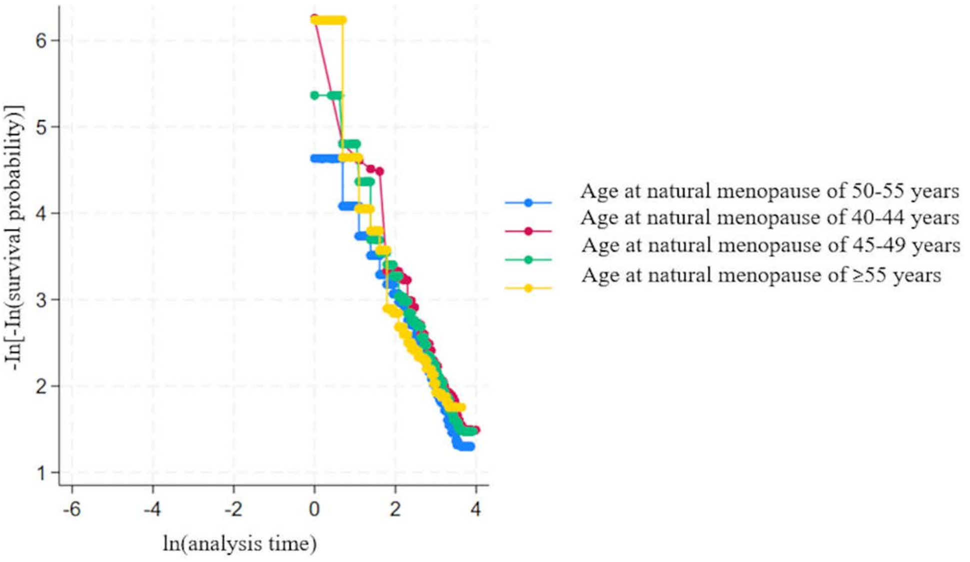

Fig 3. Cox Proportional Hazard assumption using adjusted log-log plots.

https://doi.org/10.1371/journal.pone.0324635.g003

This study had a high degree of generalizability given the large sample size and use of weighting to represent the Canadian population. Validity and precision were enhanced by the inclusion of detailed information on sociodemographic, health-related, and reproductive factors. Also, this the first study to investigate incidence of overt and not subclinical hypothyroidism by ANM categories. Nonetheless, there were several limitations to this study. Self-reported incidence of hypothyroidism and ANM could lead to recall and misclassification biases.. A study by Verschoor and Tamim indicated that self-reported age at menopause is reliable with a high degree of accuracy [27] Furthermore, hypothyroidism after menopause is difficult to diagnose given the similarities of symptoms between menopause and hypothyroidism [3,6]. Also, typical symptoms of hypothyroidism are less evident in the older population, and their symptoms are sometimes confused for other co-morbid conditions [44] or to aging [45]. It is also possible that some women with subclinical hypothyroidism may have reported it as overt hypothyroidism. These factors may have resulted in over or underestimation of the true incidence of hypothyroidism. In the Tracking cohort, BMI was self-reported. Overweight and obese populations tend to underestimate their BMI levels [46]. However, having accurate BMI results would probably have strengthen the association between higher BMI levels and hypothyroidism. The study would have been stronger if it had included actual measurements of thyroid and sex hormone levels, rather than relying solely on self-reported doctor diagnosis. Furthermore, the generalizability of the findings may be impacted by the CLSA exclusion criteria and the fact that 94.4% of the sample being of White ethnicity. Additionally, there is risk of some violations of the proportional hazard assumption, but this was minimized by using robust standard errors.

Conclusion

Our study found no evidence of any statistically significant associations between ANM and risk of hypothyroidism in postmenopausal women. Higher BMI levels have shown to be associated with hypothyroidism. A longitudinal examination of hypothyroidism and BMI could offer deeper insight into the hormonal and metabolic factors involved in the disease development. Future studies could benefit from incorporating actual thyroid and sex hormone measurements for clinical confirmation of the disease to provide a more accurate assessment of the relationship between age at menopause and hypothyroidism.

Acknowledgments

"This research has been conducted using the CLSA Baseline Tracking Dataset Version 4.0, Baseline Comprehensive Dataset Version 7.0, Follow-up 1 Tracking Dataset Version 2.3, Follow-up 1 Comprehensive Dataset Version 3.2, Follow-up 2 Tracking Dataset Version 1.0, Follow-up 2 Comprehensive Dataset Version 1.0 and participant status version 3.0, under Application Number 2206014."

'The opinions expressed in this manuscript are the author's own and do not reflect the views of the Canadian Longitudinal Study on Aging.'

Author contributions

Conceptualization: Durmalouk Kesibi, Hala Tamim.

Data curation: Durmalouk Kesibi.

Formal analysis: Durmalouk Kesibi, Michael Rotondi, Heather Edgell, Hala Tamim.

Funding acquisition: Michael Rotondi.

Methodology: Durmalouk Kesibi, Hala Tamim.

Software: Durmalouk Kesibi.

Supervision: Michael Rotondi, Heather Edgell, Hala Tamim.

Validation: Durmalouk Kesibi.

Visualization: Durmalouk Kesibi.

Writing – original draft: Durmalouk Kesibi.

Writing – review & editing: Durmalouk Kesibi, Michael Rotondi, Heather Edgell, Hala Tamim.

References

1. Dunn D, Turner C. Hypothyroidism in Women. Nurs Womens Health. 2016;20(1):93–8. https://doi.org/10.1016/j.nwh.2015.12.002 PMID: 26902444
2. Shahid MA, Ashraf MA, Sharma S. Physiology, Thyroid Hormone [Internet]. Treasure Island (FL): StatPearls Publishing; 2022 [cited 2022 Jul 12]. Available from: http://www.ncbi.nlm.nih.gov/books/NBK500006/
3. del Ghianda S, Tonacchera M, Vitti P. Thyroid and menopause. Climacteric. 2014;17(3):225–34. https://doi.org/10.3109/13697137.2013.838554 PMID: 23998691
4. Qiu Y, Hu Y, Xing Z, Fu Q, Zhu J, Su A. Birth control pills and risk of hypothyroidism: a cross-sectional study of the National Health and Nutrition Examination Survey, 2007-2012. BMJ Open. 2021;11(6):e046607. https://doi.org/10.1136/bmjopen-2020-046607 PMID: 34162647
5. Vanderpump MP, Tunbridge WM, French JM, Appleton D, Bates D, Clark F, et al. The incidence of thyroid disorders in the community: a twenty-year follow-up of the Whickham Survey. Clin Endocrinol (Oxf). 1995;43(1):55–68. https://doi.org/10.1111/j.1365-2265.1995.tb01894.x PMID: 7641412
6. Mintziori G, Veneti S, Poppe K, Goulis DG, Armeni E, Erel CT, et al. EMAS position statement: Thyroid disease and menopause. Maturitas. 2024;185:107991. https://doi.org/10.1016/j.maturitas.2024.107991 PMID: 38658290
7. Benencia H, Ropelato MG, Rosales M, Mesch V, Siseles N, Boero L, et al. Thyroid profile modifications during oral hormone replacement therapy in postmenopausal women. Gynecol Endocrinol. 1998;12(3):179–84. https://doi.org/10.3109/09513599809015542 PMID: 9675564
8. Torre F, Calogero AE, Condorelli RA, Cannarella R, Aversa A, La Vignera S. Effects of oral contraceptives on thyroid function and vice versa. J Endocrinol Invest. 2020;43(9):1181–8. https://doi.org/10.1007/s40618-020-01230-8 PMID: 32219692
9. Biondi B, Cappola AR, Cooper DS. Subclinical Hypothyroidism: A Review. JAMA. 2019;322(2):153–60. https://doi.org/10.1001/jama.2019.9052 PMID: 31287527
10. Goodwin TM, Montoro MN, Muderspach LI, Paulson RJ, Roy S. Management of Common Problems in Obstetrics and Gynecology. Wiley. 2010. https://doi.org/10.1002/9781444323030
11. Dahiya K, Dalal D, Malhotra V, Aggarwal S, Dhankhar R. Effect of thyroid status on reproductive hormones and biochemical parameters in post-menopausal women. Indian J Clin Biochem. 2022;34(Suppl 1):S130.
12. Suchartwatnachai C, Thepppisai U, Jirapinyo M. Screening for hypothyroidism at a menopause clinic. Int J Gynaecol Obstet. 2002;77(1):39–40. https://doi.org/10.1016/s0020-7292(01)00558-6 PMID: 11929657
13. Uygur MM, Yoldemir T, Yavuz DG. Thyroid disease in the perimenopause and postmenopause period. Climacteric. 2018;21(6):542–8. https://doi.org/10.1080/13697137.2018.1514004 PMID: 30296186
14. Monterrosa-Castro Á, Monterrosa-Blanco A, Sánchez-Zarza S. Possible association between subclinical hypothyroidism and age at menopause in Colombian women. Gynecol Endocrinol. 2024;40(1):2334798. https://doi.org/10.1080/09513590.2024.2334798 PMID: 38590105
15. Szeliga A, Calik-Ksepka A, Maciejewska-Jeske M, Grymowicz M, Smolarczyk K, Kostrzak A, et al. Autoimmune Diseases in Patients with Premature Ovarian Insufficiency-Our Current State of Knowledge. Int J Mol Sci. 2021;22(5):2594. https://doi.org/10.3390/ijms22052594 PMID: 33807517
16. Andersen SL, Carlé A, Olsen J, Laurberg P. Hypothyroidism incidence in and around pregnancy: a Danish nationwide study. Eur J Endocrinol. 2016;175(5):387–93. https://doi.org/10.1530/EJE-16-0446 PMID: 27511824
17. Kotopouli M, Stratigou T, Antonakos G, Christodoulatos GS, Karampela I, Dalamaga M. Early menarche is independently associated with sub-clinical hypothyroidism: a cross-sectional study. Hormone Molecular Biology and Clinical Investigation. 2019;38(1). https://doi.org/10.1515/hmbci-2018-0079
18. Frank P, Kay CR. Incidence of thyroid disease associated with oral contraceptives. Br Med J. 1978;2(6151):1531. https://doi.org/10.1136/bmj.2.6151.1531 PMID: 728709
19. Strieder TGA, Prummel MF, Tijssen JGP, Endert E, Wiersinga WM. Risk factors for and prevalence of thyroid disorders in a cross-sectional study among healthy female relatives of patients with autoimmune thyroid disease. Clin Endocrinol (Oxf). 2003;59(3):396–401. https://doi.org/10.1046/j.1365-2265.2003.01862.x PMID: 12919165
20. Vestergaard P, Rejnmark L, Weeke J, Hoeck HC, Nielsen HK, Rungby J, et al. Smoking as a risk factor for Graves' disease, toxic nodular goiter, and autoimmune hypothyroidism. Thyroid. 2002;12(1):69–75. https://doi.org/10.1089/105072502753451995 PMID: 11838733
21. Brand JS, van der Schouw YT, Onland-Moret NC, Sharp SJ, Ong KK, Khaw K-T, et al. Age at menopause, reproductive life span, and type 2 diabetes risk: results from the EPIC-InterAct study. Diabetes Care. 2013;36(4):1012–9. https://doi.org/10.2337/dc12-1020 PMID: 23230098

22. Svejme O, Ahlborg HG, Nilsson J-Å, Karlsson MK. Early menopause and risk of osteoporosis, fracture and mortality: a 34-year prospective observational study in 390 women. BJOG. 2012;119(7):810–6. https://doi.org/10.1111/j.1471-0528.2012.03324.x PMID: 22531019
23. Bove R, Chitnis T, Houtchens M. Menopause in multiple sclerosis: therapeutic considerations. J Neurol. 2014;261(7):1257–68. https://doi.org/10.1007/s00415-013-7131-8 PMID: 24101131
24. Santin AP, Furlanetto TW. Role of estrogen in thyroid function and growth regulation. J Thyroid Res. 2011;2011:875125. https://doi.org/10.4061/2011/875125 PMID: 21687614
25. Raina P, Wolfson C, Kirkland S, Griffith LE, Balion C, Cossette B, et al. Cohort Profile: The Canadian Longitudinal Study on Aging (CLSA). Int J Epidemiol. 2019;48(6):1752–1753j. https://doi.org/10.1093/ije/dyz173 PMID: 31633757
26. Raina PS, Wolfson C, Kirkland SA, Griffith LE, Oremus M, Patterson C, et al. The Canadian longitudinal study on aging (CLSA). Can J Aging. 2009;28(3):221–9. https://doi.org/10.1017/S0714980809990055 PMID: 19860977
27. Verschoor CP, Tamim H. Frailty Is Inversely Related to Age at Menopause and Elevated in Women Who Have Had a Hysterectomy: An Analysis of the Canadian Longitudinal Study on Aging. J Gerontol A Biol Sci Med Sci. 2019;74(5):675–82. https://doi.org/10.1093/gerona/gly092 PMID: 29688443
28. Mondul AM, Rodriguez C, Jacobs EJ, Calle EE. Age at natural menopause and cause-specific mortality. Am J Epidemiol. 2005;162(11):1089–97. https://doi.org/10.1093/aje/kwi324 PMID: 16221806
29. Khan D, Rotondi M, Edgell H, Tamim H. The association between shift work exposure and the variations in age at natural menopause among adult Canadian workers: results from the Canadian Longitudinal Study on Aging (CLSA). Menopause. 2022;29(7):795–804. https://doi.org/10.1097/GME.0000000000001981 PMID: 35324545
30. Royston P, White I. Multiple Imputation by Chained Equations (MICE): Implementation inStata. J Stat Soft. 2011;45(4). https://doi.org/10.18637/jss.v045.i04
31. Canadian Longitudinal Study on Aging. Data support documentation [Internet]. [cited 2024 Sep 23]. Available from: https://www.clsa-elcv.ca/researchers/data-support-documentation
32. Arafah BM. Increased need for thyroxine in women with hypothyroidism during estrogen therapy. N Engl J Med. 2001;344(23):1743–9. https://doi.org/10.1056/NEJM200106073442302 PMID: 11396440
33. Mandel SJ, Larsen PR, Seely EW, Brent GA. Increased need for thyroxine during pregnancy in women with primary hypothyroidism. N Engl J Med. 1990;323(2):91–6. https://doi.org/10.1056/NEJM199007123230204 PMID: 2359428
34. Ceresini G, Milli B, Morganti S, Maggio M, Bacchi-Modena A, Sgarabotto MP, et al. Effect of estrogen therapy for 1 year on thyroid volume and thyroid nodules in postmenopausal women. Menopause. 2008;15(2):326–31. https://doi.org/10.1097/gme.0b013e318148b83e PMID: 17998883
35. Marqusee E, Braverman LE, Lawrence JE, Carroll JS, Seely EW. The effect of droloxifene and estrogen on thyroid function in postmenopausal women. J Clin Endocrinol Metab. 2000;85(11):4407–10. https://doi.org/10.1210/jcem.85.11.6975 PMID: 11095487
36. Xu W, Huang Y, Ma L, Chen P, Li S, Chu K, et al. Clinical observation of menopause hormone therapy in postmenopausal women with euthyroid and mild subclinical hypothyroidism. BMC Endocr Disord. 2023;23(1):21. https://doi.org/10.1186/s12902-023-01269-7 PMID: 36691016
37. Fisher JS, D'Angelo SA. Stimulatory and inhibitory action of estradiol on TSH secretion. Endocrinology. 1971;88(3):687–91. https://doi.org/10.1210/endo-88-3-687 PMID: 5541304
38. Sanyal D, Raychaudhuri M. Hypothyroidism and obesity: An intriguing link. Indian J Endocrinol Metab. 2016;20(4):554–7. https://doi.org/10.4103/2230-8210.183454 PMID: 27366725
39. Song R-H, Wang B, Yao Q-M, Li Q, Jia X, Zhang J-A. The Impact of Obesity on Thyroid Autoimmunity and Dysfunction: A Systematic Review and Meta-Analysis. Front Immunol. 2019;10:2349. https://doi.org/10.3389/fimmu.2019.02349 PMID: 31681268
40. Bastemir M, Akin F, Alkis E, Kaptanoglu B. Obesity is associated with increased serum TSH level, independent of thyroid function. Swiss Med Wkly. 2007;137(29–30):431–4. https://doi.org/10.4414/smw.2007.11774 PMID: 17705106
41. Nyrnes A, Jorde R, Sundsfjord J. Serum TSH is positively associated with BMI. Int J Obes (Lond). 2006;30(1):100–5. https://doi.org/10.1038/sj.ijo.0803112 PMID: 16189501
42. Verma A, Jayaraman M, Kumar HKVS, Modi KD. Hypothyroidism and obesity. Cause or effect?. Saudi Med J. 2008;29(8):1135–8. PMID: 18690306
43. Laurberg P, Knudsen N, Andersen S, Carlé A, Pedersen IB, Karmisholt J. Thyroid function and obesity. Eur Thyroid J. 2012;1(3):159–67. https://doi.org/10.1159/000342994 PMID: 24783015
44. Leng O, Razvi S. Hypothyroidism in the older population. Thyroid Res. 2019;12:2. https://doi.org/10.1186/s13044-019-0063-3 PMID: 30774717
45. Mariotti S, Franceschi C, Cossarizza A, Pinchera A. The aging thyroid. Endocr Rev. 1995;16(6):686–715. https://doi.org/10.1210/edrv-16-6-686 PMID: 8747831
46. Quan S, Menec V, O'Connell M, Cloutier D, Newall N, Tate R, et al. Prevalence of obesity and elevated body mass index along a progression of rurality: A cross-sectional study - The Canadian Longitudinal Study on Aging. Can J Rural Med. 2022;27(4):148–57. https://doi.org/10.4103/cjrm.cjrm_55_21 PMID: 36254938
